# STRIDE: A Sequencing Depth Robust Metric for Sparse Chromosome Conformation Capture Data Comparison

**DOI:** 10.1002/advs.78529

**Published:** 2026-10-28

**Authors:** Bingxiang Xu, Xiaomeng Gao, Weichu Tao, Zhihua Zhang, Feifei Li

**Affiliations:** ^1^ Key Laboratory of Hebei Province for Molecular Biophysics Institute of Biophysics School of Health Science & Biomedical Engineering Hebei University of Technology Tianjin China; ^2^ School of Exercise and Health Shanghai University of Sport Shanghai China; ^3^ Department of Cellular and Molecular Medicine University of California San Diego School of Medicine La Jolla California USA; ^4^ China National Center for Bioinformation Beijing China; ^5^ Beijing Institute of Genomics Chinese Academy of Sciences Beijing China; ^6^ School of Life Science University of Chinese Academy of Sciences Beijing China; ^7^ Division of Cell, Developmental and Integrative Biology School of Medicine South China University of Technology Guangzhou China

**Keywords:** chromatin conformation capture, mean first passage time, sequencing depth, single‐cell Hi‐C

## Abstract

High‐throughput chromatin conformation capture technologies, such as Hi‐C and its derivatives, have revolutionized the study of 3D genome organization. However, the robustness of contact‐frequency‐based analyses to sequencing depth remains a major challenge, particularly for sparse datasets such as single‐cell Hi‐C. To overcome this challenge, we present a novel computational framework that employs the mean first passage time (MFPT) from Markov chain theory to convert chromatin contact matrices into a graph‐based effective distance representation. We demonstrate that the MFPT representation is empirically more robust to sequencing‐depth reduction and supports reliable downstream 3D genomic analyses, including TAD and chromatin loop detection, differential chromatin contact analysis, as well as the imputation and representation of sparse single‐cell Hi‐C data. Building on this representation, we developed STRIDE (spatial topological representation of interaction distance evaluation), a library similarity metric that does not require dataset‐specific parameter optimization. STRIDE is robust to sparse and noisy data and shows improved resilience to technical variation, thereby facilitating accurate identification of cellular states and developmental trajectories in the datasets analyzed. Together, the MFPT representation and STRIDE provide a robust computational framework for sequencing‐depth‐resilient analysis of 3D genome organization.

## Graphical abstract

A mean first passage time (MFPT)‐based representation transforms sparse Hi‐C contact maps into robust topological representations, preserving biologically meaningful chromatin features and facilitating robust analysis of TADs, chromatin loops, differential chromatin contacts, and sparse single‐cell Hi‐C data. MFPT‐derived STRIDE enables reliable comparison of Hi‐C libraries across technical and sequencing depth variation.

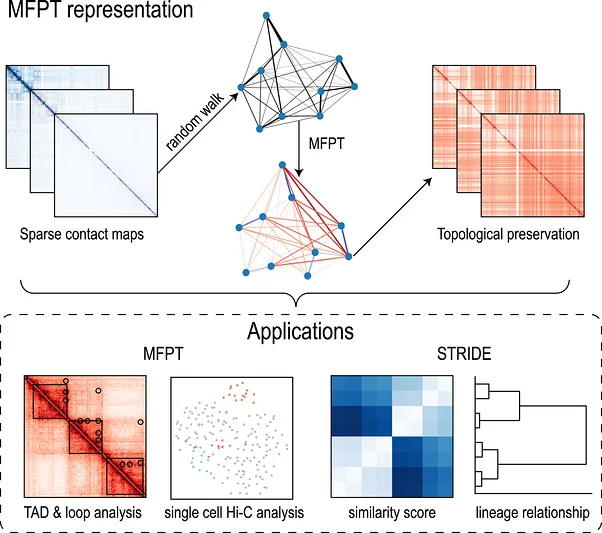

## 1 Background

High‐throughput chromatin conformation capture techniques, including Hi‐C, ChIA‐PET, and HiChIP/PLAC‐Seq, have dramatically advanced our understanding of 3D genome architecture by enabling high‐resolution, genome‐wide mapping of chromosomal interactions [1, 2, 3, 4]. These approaches allow analyses across multiple scales—from megabase‐sized A/B compartments to smaller topologically associating domains (TADs) and chromatin loops—and have revealed the dynamic reorganization of 3D genome structure during development and in response to environmental cues [5, 6, 7, 8, 9, 10, 11].

The 2D nature of Hi‐C contact matrices requires sequencing depth to scale quadratically with desired resolution [12, 13]. For instance, producing reliable 1‐kb resolution maps in human GM12878 cells demands approximately 5 billion valid read pairs [14]. Such depths are often unattainable, particularly in single‐cell Hi‐C, where individual cells typically yield no more than 1 million read pairs [15, 16, 17]. Consequently, the majority of Hi‐C datasets remain sparse and undersaturated, introducing substantial stochastic noise. This noise propagates through downstream analyses, compromising the reproducibility of library similarity assessments, differential interaction calling, and detection of chromatin features [16]. Despite extensive efforts to mitigate sparsity in specific downstream tasks, current approaches remain largely application‐specific [18, 19]. A general, representation‐level framework for improving the robustness of Hi‐C analyses to sequencing‐depth reduction is still lacking.

Assessing similarity (or distance) between Hi‐C libraries is a fundamental task in 3D genome analysis. Existing methods employ diverse strategies to reduce noise and to counteract the strong correlation between raw contact frequencies and genomic distance. These include local smoothing approaches (e.g., HiCRep [20] and QuaSAR [21]), spectral decomposition (HiC‐spector [22]), and random‐walk‐based imputation with concurrent noise reduction (genomeDISCO [23]). While these algorithms often show reduced robustness when comparing libraries with substantially different sequencing depths, they typically address this limitation by randomly down‐sampling libraries to equalize sequencing depth, without incorporating more effective approaches [20, 23]. Random down‐sampling inevitably discards large amounts of valuable data, introduces additional stochastic variability, and complicates comparisons among multiple libraries.

Growing evidence shows that contact frequencies in Hi‐C matrices are strongly correlated, particularly between adjacent or spatially proximal genomic bins [1, 24]. This property has led to the use of Markov chain (random walk) models for unsupervised smoothing and imputation of sparse Hi‐C data [23, 25, 26]. However, existing approaches typically restrict walks, either explicitly or implicitly, to a limited number of steps [23, 26], thereby limiting their exploration to local neighborhoods and constraining overall denoising performance.

The mean first passage time (MFPT)—defined as the expected number of steps for a random walk to reach a target bin from a given starting bin—overcomes this limitation. Crucially, MFPT estimation is dominated by high‐probability paths (i.e., those supported by frequent contacts), which are expected to remain relatively stable even when sequencing depth is substantially reduced. Consequently, MFPT offers a natural, graph‐based representation of inter‐bin relationships that is empirically robust to sequencing depth (Figure S1A).

Leveraging these insights, we developed a novel computational framework that transforms chromatin contact matrices into a robust, graph‐based representation using MFPT. This transformation exploits correlations among proximal bins while remaining empirically robust to sequencing‐depth reduction. Building on this foundation, we introduce STRIDE (spatial topological representation of interaction distance evaluation), a metric that does not require dataset‐specific parameter optimization for quantifying library (dis)similarity. We show that STRIDE is robust to fluctuations in sequencing depth and technical noise, yet retains sensitivity to biologically meaningful differences in the datasets analyzed. Furthermore, MFPT enables the imputation and unsupervised embedding of single‐cell Hi‐C data, providing a robust representation for downstream single‐cell Hi‐C analyses.

## 2 Results

### 2.1 MFPT Acts as an Empirically Sequencing‐Depth‐Robust Representation of the Contact Data

The STRIDE algorithm exploits the sequencing‐depth‐insensitive properties of mean first passage time (MFPT) representations derived from contact matrices. In contrast to contact frequency‐based methods, MFPT quantifies the graph‐based effective relationship between any pair of genomic bins as the average number of steps required for a random walk to travel between them, where transition probabilities are defined by normalized contact frequencies. Specifically, a balanced Hi‐C contact matrix is interpreted as a transition matrix for a finite‐state Markov chain (random walk process). For irreducible and positive recurrent chains, the MFPT between bins reflects their effective connectivity in the chromatin interaction network, with transitions dominated by high‐probability paths (strong contacts). These dominant paths are expected to remain relatively stable under down‐sampling, thereby providing two practical advantages: (i) greatly reduced variability to sequencing depth, as rare or absent contacts contribute minimally to MFPT, and (ii) preservation of spatial relationships even for bin pairs with zero observed contacts through indirect connectivity (Figure S1A). These properties enable robust inference of chromatin organization from sparse data (see Methods for details).

To assess the empirical robustness of the MFPT representation to sequencing depth, we progressively down‐sampled a deeply sequenced GM12878 dataset [14] to fractions of 1/2, 1/4, 1/8, 1/16 and 1/32. At the lowest depth tested (≈80 million cis contacts), more than 72.5% of bin pairs at 10‐kb resolution had zero contacts, and ≈66% of annotated loops were no longer detectable in raw contact maps—a sparsity level comparable to that of pooling about 1,000–2,000 single cells [26]. Whereas raw contact maps rapidly became dominated by noise upon down‐sampling (Figure 1A), MFPT matrices retained their overall global structure even at the lowest depth (Figure 1B). Quantitatively, MFPT matrices consistently exhibited higher signal‐to‐noise ratios (SNR) across all chromosomes and sampling fractions (Figure 1C; Supporting Information).

**FIGURE 1:**
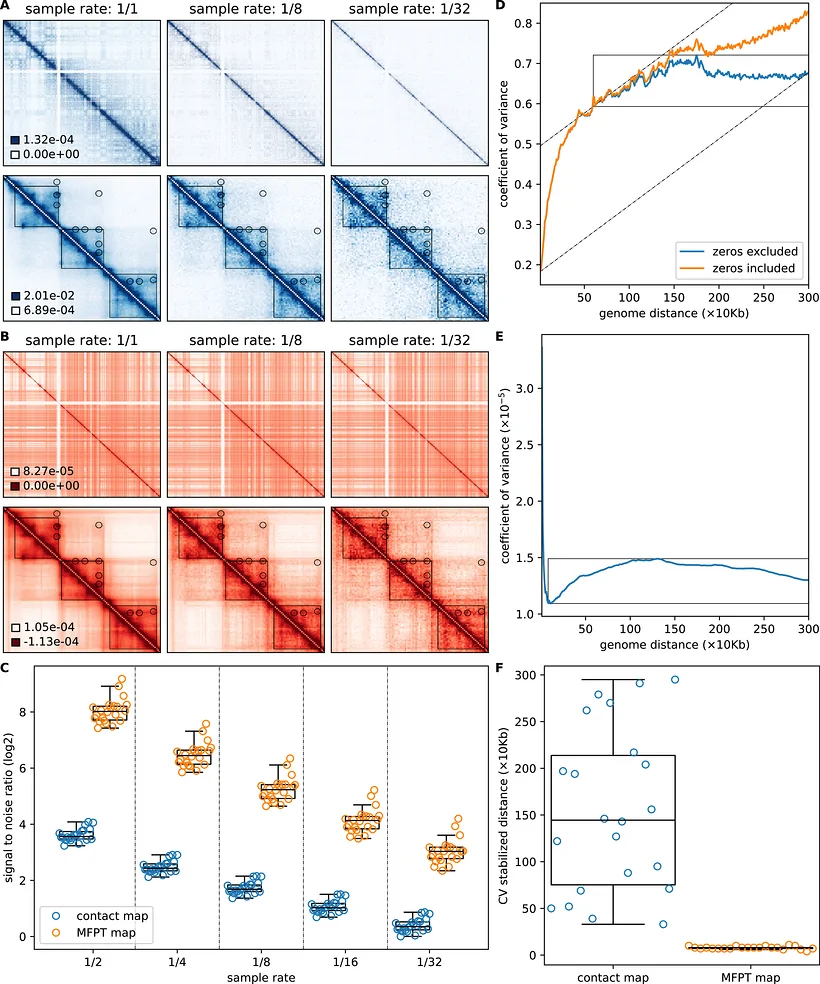
MFPT matrices preserve chromatin‐contact patterns at reduced sequencing depths. (A) Raw contact maps and (B) corresponding MFPT matrices for chromosome 6 from the GM12878 dataset at the indicated downsampling rates. All matrices are shown at 10‐kb resolution. Top panels display the whole chromosome (every tenth bin shown); bottom panels show a zoomed region (chr6:42.50–43.50 Mb) with TAD boundaries and chromatin loops identified from the full‐depth dataset marked. For clarity, only the full dataset and the 1/8 and 1/32 downsampling rates are shown. (C) Signal‐to‐noise ratio (SNR) of contact maps and MFPT matrices across all chromosomes and sequencing depths. (D, E) Coefficient of variation (CV) of contact frequencies (D) and MFPT values (E) as a function of genomic distance. In (D), CV was calculated with (yellow) or without (blue) zero‐contact bin pairs. Boxes indicate the distance ranges where CV reaches a stable plateau. (F) Chromosome‐wise distributions of the genomic distances at which CV reaches a stable plateau for contact maps and MFPT matrices.

We next examined the dependence of variability on genomic distance. While the coefficient of variation (CV) of raw contact frequencies increased markedly with genomic separation and failed to stabilize even after excluding zero‐count pairs (Figure 1D; Figure S1B), the variability of MFPT representation decreased with distance and reached a stable plateau at much shorter genomic separations (typically 40–140 kb; Figure 1E,F). This weak distance‐dependence largely eliminates the need for complex distance‐based normalization in downstream analyses.

Together, these results demonstrate that MFPT representation is empirically robust to sequencing‐depth reduction, faithfully preserves conformational patterns at low coverage, and exhibits minimal correlation between variability and genomic distance in the datasets analyzed.

### 2.2 MFPT Representation Preserves Topological Features in Low‐Coverage Chromatin Conformation Capture Libraries

The MFPT representation was expected to preserve both TAD and loop structures under reduced sequencing depth. To evaluate this, we compared the MFPT representations with conventional contact maps and maps normalized by Raichu [27], a recently developed normalization method for improving regulatory loop detection, using progressively down‐sampled GM12878 datasets.

TAD boundary preservation was evaluated using insulation scores (IS) [28]. MFPT representations produced insulation‐score profiles that remained highly stable throughout down‐sampling, comparable to those derived from conventional contact maps and Raichu‐normalized maps [27] (Figure 2A). Annotated TAD boundaries were consistently recovered across all sequencing depths, and the characteristic periodicity of TAD organization was well preserved. This observation is consistent with the well‐established robustness of TAD identification to reduced sequencing depth [29] due to its reliance on contact patterns across relatively large genomic windows around the diagonal, where contacts are enriched. The robustness was further supported by stacked contact profiles centered on annotated TAD boundaries (Figure 2B; Figure S2A,B). Although IS profiles were retained in all three representations, the underlying signals differed substantially. In conventional contact maps and Raichu‐normalized maps, both intra‐ and inter‐TAD contact signals progressively weakened during down‐sampling (Figure S2A,B), whereas the MFPT representation largely preserved these relative contact patterns across TAD boundaries (Figure 2B). We further evaluated whether this robustness extended to de novo TAD identification using a simplified insulation‐score‐based caller (Supporting Information) derived from our previous study [30]. Across sequencing depths, MFPT yielded a stable number of TADs and maintained >80% consistency with full‐depth calls, with slightly higher consistency than conventional contact maps (Figure S2C). The resulting MFPT‐derived boundaries also showed similar or, in some cases, slightly greater overlap with CTCF, RAD21, and SMC3 ChIP‐seq peaks than contact map ‐ derived boundaries (Table S1; Figure S2D). Together, these results suggest that the MFPT representation preserves biologically meaningful TAD‐associated contact patterns under reduced sequencing depth, and that this preservation could in turn support de novo TAD identification.

**FIGURE 2:**
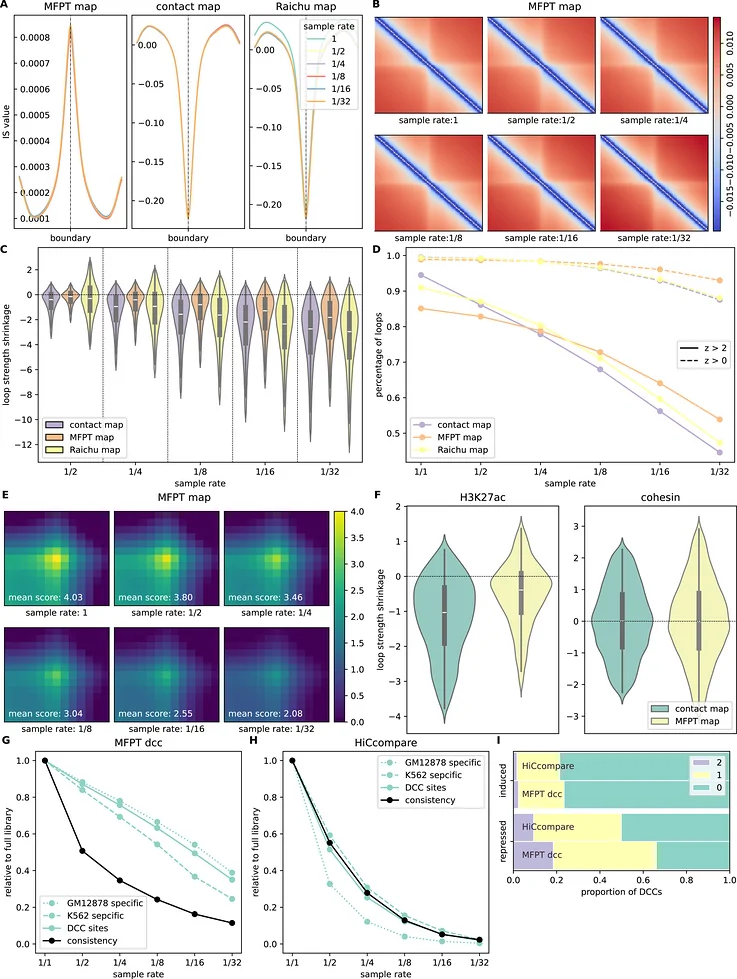
MFPT representation robustly preserves topological features in low‐coverage Hi‐C and HiChIP datasets. (A) Stacked insulation‐score profiles centered on annotated TAD boundaries from MFPT representations, conventional contact maps and the Raichu maps at the indicated down‐sampling depths. (B) Average MFPT profiles centered on annotated TAD boundaries. The upper‐right and lower‐left corners correspond to inter‐TAD regions. (C) Distributions of loop intensity (Z‐scores) for reference loops across sequencing depths. (D) Fractions of reference loops remaining above the indicated Z‐score thresholds across sequencing depths. (E) Aggregate peak analysis (APA) of reference loops calculated from MFPT representations across down‐sampling levels. Numbers indicate the average Z‐score of loops involved. (F) Distributions of loop intensity (Z‐scores) for loops across sequencing depths in the HiChIP datasets. (G, H) Numbers of DCCs identified, including K562‐ and GM12878‐specific DCCs, together with their consistency across down‐sampling levels for the MFPT‐based method (G) and HiCcompare (H). Consistency was quantified as the geometric mean of the Jaccard indices between DCCs identified from each down‐sampled library and those identified from the corresponding full‐depth library in the two cell types. (I) Proportions of DCCs with zero, one, or two anchors overlapping RAD21 binding peaks in the HCT116 RAD21 knockdown experiment.

MFPT preserved contact enrichment at chromatin loops more effectively. To avoid potential bias in loop identification, we used an independent reference set supported by ChIA‐PET [31] and HiChIP [3, 32] experiments (Methods; Table S1, 83865 loops at 10‐kb resolution in total). Relative to those in the corresponding full‐depth library, loop‐associated signals decayed substantially more slowly in MFPT representations than in either conventional contact maps or Raichu‐normalized maps. On average, loop Z‐scores (Methods) decreased by 0.5532 (95% CI, 0.5016–0.6048) following each two‐fold reduction in sequencing depth, compared with 0.7456 (95% CI, 0.6901–0.8011) for conventional contact maps and 0.8812 (95% CI, 0.8151–0.9473) for Raichu‐normalized maps (Figure 2C). Consequently, MFPT representations consistently retained a larger fraction of significant loops (*Z* > 0 or *Z* > 2) once sequencing depth decreased to approximately one‐quarter to one‐eighth of the original library, and this advantage became progressively greater at lower sequencing depths (Figure 2D).

These observations were further supported by genome‐wide aggregate peak analysis (APA). APA showed concentrated loop enrichment at all sequencing depths, with the enrichment signal decaying more slowly in the MFPT representation. (Figure 2E; Figure S2E,F) [33]. Moreover, loop Z‐scores derived from the MFPT representation consistently distinguished validated loops from matched negative interactions (Methods) with higher accuracy than conventional contact maps or Raichu‐normalized maps, yielding substantially higher AUC values at reduced sequencing depths (Figure S2G). Together, these results demonstrate that the MFPT representation preserves biologically meaningful loop‐associated contact signals under sequencing‐depth reduction.

This robustness extended to biologically critical functional loops. For example, an H3K27ac HiChIP‐validated promoter–promoter loop between CCDC58 and FAM162A [3] was clearly detectable in full‐depth Hi‐C contact maps but became undetectable below ≈1/8 sampling (Figure S2H). In striking contrast, the same loop remained highly significant across all tested depths in the MFPT representation (Figure S2I).

This improved preservation of loop‐associated signals supports de novo loop identification. We applied a simplified MFPT‐based loop caller (Supporting Information) and compared its performance with mustache [34] applied to conventional balanced contact maps and Raichu‐normalized maps. Across sequencing depths, loops were ranked by statistical significance and evaluated against the independent ChIA‐PET/HiChIP reference set. At high sequencing depths, the MFPT‐based approach recovered more independently supported loops for the same number of top‐ranked calls. As sequencing depth decreased, the validation rates became increasingly similar among the three approaches (Figure S2J). However, at the same FDR threshold, MFPT continued to identify a larger number of loops, with the additional calls still containing independently supported interactions (Figure S2J).

We next evaluated the robustness of the MFPT representation in inherently sparse HiChIP datasets, in which contact‐map‐based loop detection is particularly sensitive to sequencing depth. We analyzed H3K27ac HiChIP (≈7% of the corresponding Hi‐C depth [3]) and cohesin HiChIP (≈4%) from GM12878 cells [32], further down‐sampling both by 50% to mimic the lowest‐coverage of public HiChIP libraries [35]. Raichu was not included because its applicability to HiChIP data has not been established. Consistent with the Hi‐C results, MFPT representations better preserved loop‐associated contact signals under sequencing‐depth reduction. In the H3K27ac HiChIP dataset, loop Z‐scores decreased substantially more slowly than those in conventional contact maps, whereas in the cohesin HiChIP dataset, MFPT yielded comparably stable representations (Figure 2F). Moreover, MFPT consistently retained a larger fraction of significant loops after down‐sampling (H3K27ac: 88.3%→83.5% for MFPT vs 86.6%→73.5% for contact map; cohesin: 70.4%→71.7% for MFPT vs 57.8%→57.6% for contact map), demonstrating that the MFPT representation also preserves biologically meaningful contact signals in sparse HiChIP libraries.

Finally, the MFPT representation enables the differential chromatin conformation (DCC) analysis. To show this, we performed a proof‐of‐concept analysis using the MFPT representation between GM12878 and K562 cells at 10‐kb resolution (see Methods for algorithmic details). Compared with HiCcompare [36], a state‐of‐the‐art DCC algorithm, the MFPT‐based approach consistently identified more DCCs while maintaining higher consistency with the full‐depth libraries during down‐sampling (Figure 2G). HiCcompare rapidly lost sensitivity below approximately one‐quarter to one‐eighth of the original sequencing depth and detected almost no K562‐specific DCCs (Figure 2H). The biological relevance of the identified DCCs was further supported by the RAD21 knockdown experiment in HCT116 cells [37]. RAD21 binding was expected to be enriched at the anchors of interactions weakened after RAD21 depletion. MFPT‐based DCCs showed stronger enrichment than those identified by HiCcompare for weakened DCCs after RAD21 depletion, whereas the two methods performed comparably for the strengthened DCCs. Overall, the MFPT‐based results were more consistent with the expected biological consequences of RAD21 depletion (Figure 2I).

These results establish that the MFPT representation robustly preserves key topological features—TADs and loop‐associated signals—across a wide range of sequencing depths and experimental formats. The MFPT representation also provides a promising foundation for differential chromatin conformation analysis. Overall, our results imply MFPT as a robust representation of chromatin contact data that may empirically mitigate the impact of sequencing‐depth variation in 3D genome analyses.

### 2.3 STRIDE Robustly Measures Library Similarity across a Wide Range of Sequencing Depths

Leveraging the sequencing‐depth robustness of MFPT representations, we developed STRIDE—a metric that directly computes pairwise library dissimilarities from MFPT matrices, eliminating the need for down‐sampling or other preprocessing steps (Methods). We benchmarked STRIDE against three widely used methods (HiCRep [20], HiC‐spector [22], and genomeDISCO [23]) by converting their similarity scores to distances (Supporting Information), such that values closer to zero indicate greater similarity. Unless otherwise stated, all comparisons were performed at 50‐kb resolution.

First, we assessed the ability of each metric to distinguish pseudo‐replicates (PR), biological replicates (BR), and non‐replicates (NR) in down‐sampled GM12878 and K562 libraries [14] (1/2, 1/4, 1/8, 1/16 and 1/32 of the original sequencing depths, Table S2). A robust metric should satisfy two criteria: (i) maintain the similarity order PR > BR > NR across all depths, and (ii) show progressively decreasing similarity as sequencing depth decreases. STRIDE and genomeDISCO satisfied both criteria on every chromosome, whereas HiCRep deviated on two chromosomes (chromosome 1 and 17). In contrast, HiC‐spector violated at least one criterion on most chromosomes (Figure 3A; Figure S3) and was excluded from subsequent analyses in this section.

**FIGURE 3:**
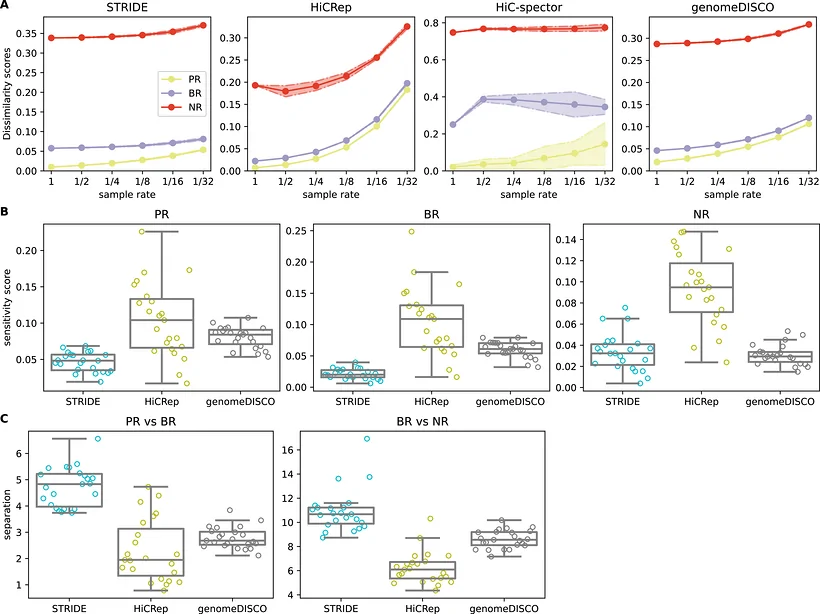
STRIDE provides consistent and robust library similarity measurements across sequencing depths. (A) Dissimilarity scores among pseudo‐replicates (PR), biological replicates (BR), and non‐replicates (NR) calculated by STRIDE, HiCRep, HiC‐spector, and genomeDISCO at the indicated down‐sampling levels. Chromosome 1 is shown as a representative example. Shaded regions indicate 95% confidence intervals estimated from 10 independent down‐sampling trials. The slopes of the curves indicate robustness of the measurements across sequencing depths. The separations between curves indicate the ability to distinguish libraries with different biological or technical relationships. (B) Sequencing‐depth sensitivity scores (lower values indicate greater robustness; see Supporting Information) for STRIDE, HiCRep, and genomeDISCO across all chromosomes. (C) Discrimination index quantifying the down‐sampling noise required to eliminate the distinctions between PR and BR (left) or BR and NR (right). Higher values reflect superior discriminative power. In (B) and (C), chromosomes 1–22 and X are plotted as individual dots from left to right.

Second, STRIDE was least affected by sequencing depth reduction, as evidenced by the most gradual change in scores across down‐sampling (Figure 3A; Figure S3). A quantitative depth‐sensitivity score (Supporting Information; Figure S4A) confirmed that STRIDE outperformed both HiCRep (Cohen's *d_z_* =   − 1.27 for PR and − 1.65 for BR, both with *p* < 1.0 × 10^−6^, paired permutation tests with 10^6^ permutations) and genomeDISCO (*d_z_* =   − 3.75 for PR and − 4.22 for BR, both with *p* < 1.0 × 10^−6^) in PR and BR comparisons (Figure 3B). In NR comparisons, STRIDE matched genomeDISCO (*d_z_* =  0.17, *p*  =  0.22) and remained significantly superior to HiCRep (*d_z_* =   − 1.96, *p* < 1.0 × 10^−6^, Figure 3B).

Third, STRIDE showed the strongest discriminative power among PR, BR and NR. We quantified the amount of down‐sampling noise required to eliminate the distinctions between PR and BR or between BR and NR (Supporting Information; Figure S4B). STRIDE consistently required the highest noise levels to disrupt these distinctions, outperforming both HiCRep (*d_z_* =  1.56 for PR‐BR and 2.62 for BR‐NR, both with *p* < 1.0 × 10^−6^) and genomeDISCO (*d_z_* =  2.89 for PR‐BR and 1.62 for BR‐NR, both with *p* < 1.0 × 10^−6^, Figure 3C).

Together, these results suggest that STRIDE is robust to sequencing‐depth variation, can reliably distinguish replicate types even under extreme sparsity, and tends to outperform existing metrics in robustness while maintaining strong discriminative performance.

### 2.4 STRIDE Is Robust to Technical Variability in Experimental and Analytical Protocols

To evaluate robustness against technical variability, we analyzed four Hi‐C libraries from hESC and IMR90 cells that differed substantially in library preparation protocols and sequencing depths [38] (Figure 4A; Table S1). Each library was processed independently using both the Juicer [39] and HiC‐Pro [40] pipelines to evaluate variability introduced by bioinformatics workflows.

**FIGURE 4:**
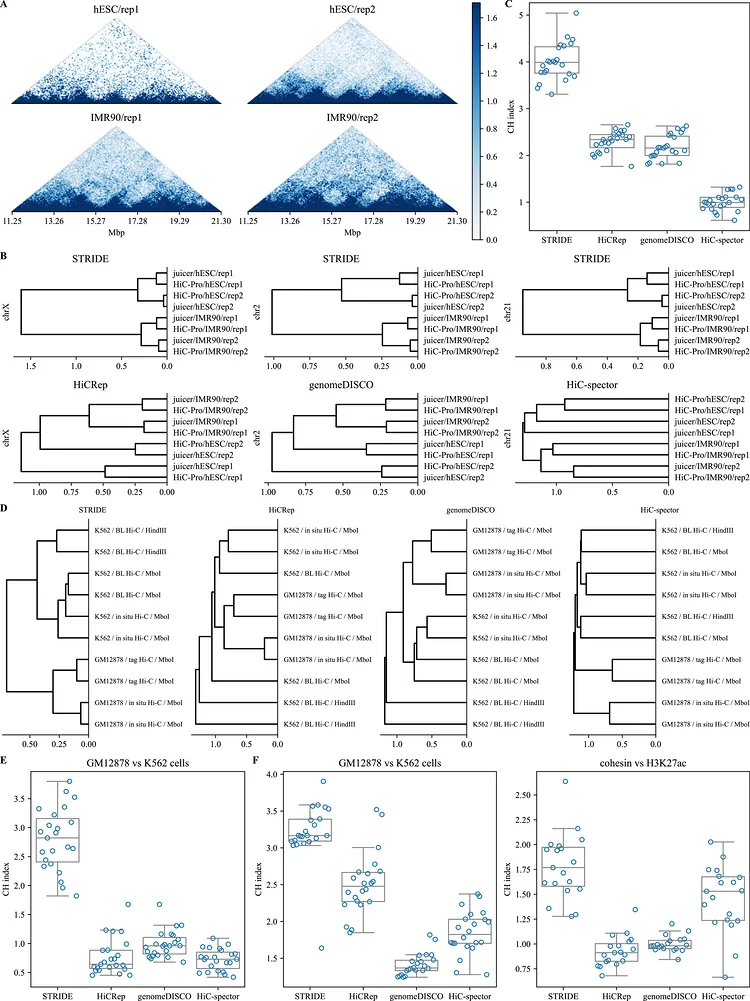
STRIDE is robust to technical variability introduced by experimental and analytical protocols. (A) Representative Hi‐C contact maps (chrX: 11.25–21.25 Mb) from hESC and IMR90 cells. (B) Hierarchical clustering of four hESC/IMR90 Hi‐C libraries generated by STRIDE, HiCRep, HiC‐spector, and genomeDISCO. Each library was processed independently using both Juicer and HiC‐Pro. Three representative chromosomes are shown. (C) Chromosome‐wise CH indices (log‐transformed) for hESC and IMR90 libraries. Higher values indicate better separation of cell types. Chromosomes 1–22 and X are shown from left to right. (D) Hierarchical clustering of independently generated GM12878 and K562 Hi‐C libraries. Chromosome 1 is shown as a representative example. (E) Chromosome‐wise CH indices for the independently generated GM12878 and K562 Hi‐C libraries. Chromosomes 1–22 and X are shown from left to right. (F) Chromosome‐wise CH indices for two HiChIP benchmarks (GM12878/K562 H3K27ac HiChIP and mESC cohesin/H3K27ac HiChIP).

An effective similarity metric should recover the clear ground truth of the relationships among these libraries. Libraries generated from the same Hi‐C dataset but processed through different bioinformatic pipelines should be preferentially clustered together, followed by biological replicates from the same cell type, whereas unrelated cell types should be separated (Figure S5A). The metric should also quantitatively cluster biologically relevant libraries as tightly as possible while keeping biologically irrelevant ones as far apart as possible. STRIDE was the only method that satisfied these criteria on every chromosome. This conclusion was supported by the similarity values (or the multidimensional scaling projection of them into the 2D space, Figure S5B; Table S3) and the hierarchical clustering results derived from them (Figure 4B; Table S3). In contrast, HiCRep and genomeDISCO separated one hESC library from the remaining hESC libraries on several chromosomes, whereas HiC‐spector frequently failed to cluster identical libraries processed by different pipelines (Figure 4B; Table S3).

STRIDE's superior ability to separate cell types was also summarized by the higher Calinski–Harabasz (CH) index calculated with the cell types as labels, which quantifies the ratio of between‐cell‐type to within‐cell‐type dispersion. STRIDE achieved a minimum CH index of 3.31 (chromosome 9, log‐transformed), exceeding the maximum values obtained by HiCRep (2.65), HiC‐spector (1.32), and genomeDISCO (2.63, all with *p* < 1.0 × 10^−6^, paired permutation tests with 10^6^ random permutations; Figure 4C).

We next extended this evaluation to independently generated GM12878 and K562 Hi‐C libraries spanning multiple experimental protocols (in situ Hi‐C [8, 14], tag Hi‐C [9], and BL Hi‐C [41]), different restriction enzymes (MboI and HindIII), and a wide range of sequencing depths (approximately 600‐fold, Table S1). STRIDE consistently grouped libraries according to cell type in 18 out of the 23 chromosomes examined. On 11 of these chromosomes, STRIDE perfectly grouped the samples hierarchically by cell type, restriction enzyme type, and experimental protocol, substantially outperforming the other methods (Figure 4D; Figure S5C; Table S4). These observations were also summarized by its higher CH index (calculated with the cell types as labels, Figure 4E). These results further demonstrate that STRIDE remains robust across diverse experimental designs and sequencing depths in independently generated datasets.

Finally, we assessed MFPT performance on inherently sparse HiChIP datasets. In the five H3K27ac HiChIP libraries from GM12878 and K562 cells [32], all four methods successfully separated the two cell types (Table S5). Nevertheless, STRIDE consistently placed biological replicates close together (by visual inspection of the MDS projected library (dis)similarity matrices, Figure S5D; Table S5), achieving the highest CH indices (taking the cell types as labels) across all chromosomes, significantly outperforming HiCRep (*d_z_* =  1.42, *p*  =  1.10  ×  10^−5^), genomeDISCO (*d_z_* =  5.07, *p* < 1.0  ×  10^−6^), and HiC‐spector (*d_z_* =  3.36, *p* < 1.0  ×  10^−6^; Figure 4F). In the more challenging mESC dataset comprising fourteen cohesin‐ and H3K27ac‐targeted HiChIP libraries generated from different numbers of mESC cells [3], STRIDE achieved near‐perfect separation of the two antibody targets, with no antibody mixing on most chromosomes. Only minor deviations were observed on chromosomes 3, 14, and 16, where the two 500K‐cell H3K27ac libraries clustered separately from the remaining H3K27ac samples (Table S6). In contrast, HiCRep misclassified the 1M‐cell cohesin library on seven chromosomes, genomeDISCO mixed H3K27ac and cohesin libraries on nine chromosomes, and HiC‐spector exhibited antibody mixing on six chromosomes. The library similarity matrices showed that STRIDE tended to cluster libraries from the same cell type and with the same initial cell number closely together, especially in the H3K27ac libraries (Figure S5E; Table S6), yielding the highest CH indices (taking the targets as labels), significantly exceeding those of HiCRep (*d_z_* =  2.86, *p* < 1.0 × 10^−6^), genomeDISCO (*d_z_* =  2.99, *p* < 1.0 × 10^−6^), and HiC‐spector (*d_z_* =  0.69, *p*  =  8.2 × 10^−4^; Figure 4F).

Together, these results demonstrate that STRIDE provides robust library similarity measurements across diverse experimental protocols and bioinformatics workflows while consistently preserving biologically meaningful differences in the datasets analyzed.

### 2.5 STRIDE Reconstructs Biologically Meaningful Lineage Relationships from Sparse Hi‐C Datasets

To evaluate whether STRIDE can reconstruct biologically meaningful lineage relationships from sparse Hi‐C datasets, we first analyzed a zebrafish hematopoietic stem and progenitor cell (HSPC) Hi‐C dataset comprising nascent, fetal, and adult HSPCs (Figure S5F). The dataset contains substantial sequencing‐depth variation (approximately 15.5‐fold, with the nascent HSPC libraries containing as few as 2.6 million valid cis contacts; Table S1) [42]. When considering only the separation between different developmental stages, STRIDE correctly reconstructed the lineage tree in 23 of the 25 chromosomes examined, whereas HiC‐spector succeeded on only six chromosomes and HiCRep and genomeDISCO failed in all chromosomes (Figure 5A; Table S7). Remarkably, STRIDE further resolved the higher degree of similarity between fetal and adult samples in 17 chromosomes, whereas none of the other methods recovered the complete hierarchy (Figure S5G). These qualitative and quantitative advantages were further supported by the CH indices (calculated with the cell types as labels), which were greater than 1 on 23 chromosomes, whereas the other three methods produced CH indices below 1 across all chromosomes (Figure 5B).

**FIGURE 5:**
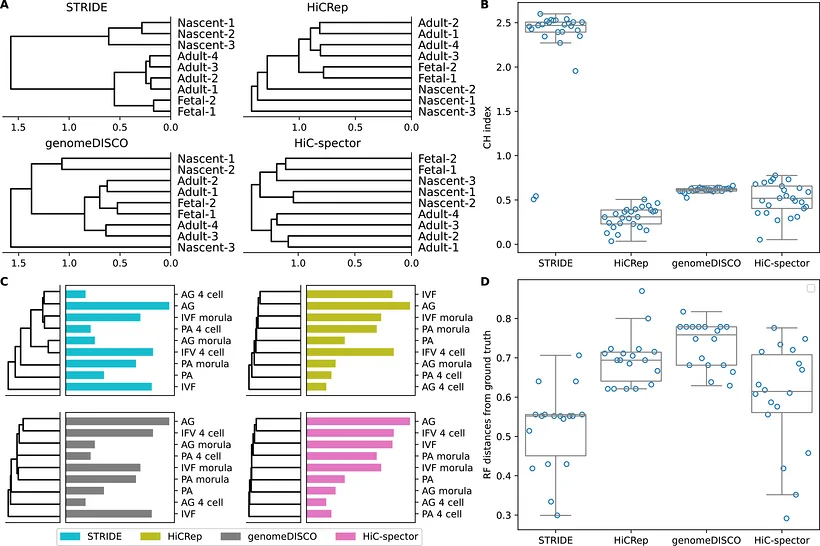
STRIDE reconstructs biologically meaningful lineage relationships from sparse Hi‐C datasets. (A) Hierarchical clustering of the zebrafish HSPC developmental Hi‐C libraries generated by STRIDE, HiCRep, genomeDISCO, and HiC‐spector. Chromosome 6 is shown as a representative example. (B) Chromosome‐wise CH indices quantifying the separation of zebrafish HSPC cell types. Chromosomes are ordered from left to right according to chromosome number. (C) Hierarchical clustering of early porcine embryonic Hi‐C libraries, including zygote, 4‐cell, and morula stages generated by IVF, PA, or AG, based on distances computed by each method. Chromosome 3 is shown as a representative example (left); bar widths on the right indicate the relative sequencing depths of individual libraries. (D) Generalized Robinson–Foulds (RF) distances measuring the difference between inferred and reference developmental trees in the porcine embryo dataset.

We next evaluated STRIDE on a low‐depth Hi‐C dataset from early porcine embryonic development (182–587 million read pairs per library) [43], including zygote, 4‐cell, and morula‐stage embryos generated by in vitro fertilization (IVF), parthenogenetic activation (PA), or androgenetic activation (AG). We evaluated lineage reconstruction using hierarchical clustering and generalized Robinson–Foulds (RF) distance, which quantifies the difference between the inferred lineage tree and the previously reported developmental hierarchy. STRIDE generated the lineage tree with a significantly smaller RF distance to the expected developmental relationships than HiCRep (*d_z_* =   − 1.41, *p* < 2.2 × 10^−5^), genomeDISCO (*d_z_* =   − 1.71, *p*  =  9.0 × 10^−6^), and HiC‐spector (*d_z_* =   − 0.49, *p*  =  0.029, paired permutation tests; Figure 5C,D; Figure S5H; Table S8). Notably, STRIDE captured the closer similarity between AG morulae and IVF 4‐cell embryos, a pattern consistent with the delayed establishment of paternal chromatin architecture during early porcine embryogenesis. None of the other metrics recovered this biologically meaningful pattern (Figure 5C; Table S8). Moreover, lineage trees inferred by HiCRep and HiC‐spector were frequently dominated by sequencing‐depth differences across chromosomes, whereas STRIDE remained largely independent of sequencing depth and preserved the expected developmental hierarchy.

Together, these results demonstrate that STRIDE combines robustness to experimental and technical variability with the ability to preserve biologically meaningful differences, enabling a more accurate reconstruction of lineage relationships from sparse Hi‐C libraries in the datasets analyzed.

### 2.6 MFPT Provides a Depth‐Robust Representation for Sparse Single‐Cell Hi‐C Analysis

Single‐cell Hi‐C analysis remains challenging due to extreme sparsity, and current approaches typically rely on imputation to recover informative representations before downstream analysis. To evaluate whether MFPT can serve as a depth‐robust representation for sparse single‐cell Hi‐C data, we integrated MFPT transformation into the scHiCluster framework [25], a dedicated scHi‐C imputation and embedding method. Specifically, we replaced the original random‐walk‐with‐restart (RWR) imputation step with MFPT transformation, removed the core binarization step in the original pipeline, while keeping the remaining workflow unchanged. This enables direct comparison between MFPT‐ and RWR‐based representations (Supporting Information).

Two independent single‐cell Hi‐C datasets were used, including an mESC cell‐cycle dataset (1572 cells) [10] and a mouse forebrain dataset (1954 cells) [11]. Overall embedding performance was comparable between MFPT and the original RWR‐based scHiCluster, with similar adjusted Rand index (ARI) values in both datasets (cell cycle: 0.3151 vs 0.3331; mouse forebrain: 0.3439 vs 0.3230; hierarchical clustering). However, MFPT consistently produced embeddings that better reflected known biological relationships. In the cell‐cycle dataset, the MFPT‐based embedding produced a more cohesive pre‐M cluster and correctly positioned it adjacent to the late‐S/G2 population, whereas the original scHiCluster embedding dispersed pre‐M cells and placed them closer to the G1 population. In addition, MFPT more clearly separated the G1 and late‐S/G2 populations, with early‐S phase cells lying between them (Figure 6A). In the mouse forebrain dataset, MFPT correctly placed the two oligodendrocyte subpopulations adjacent to each other (Figure 6B), consistent with the corresponding single‐cell RNA‐seq reference [11]. In the RWR‐based representation, these two subpopulations were located on opposite sides of the major neuronal population. In addition, microglia were more distinctly segregated from neural‐lineage populations in the MFPT representation, and neurons and astrocytes showed greater within‐group compactness (Figure 6B). These observations suggested that MFPT‐based scHiCluster provided more biologically coherent embeddings.

**FIGURE 6:**
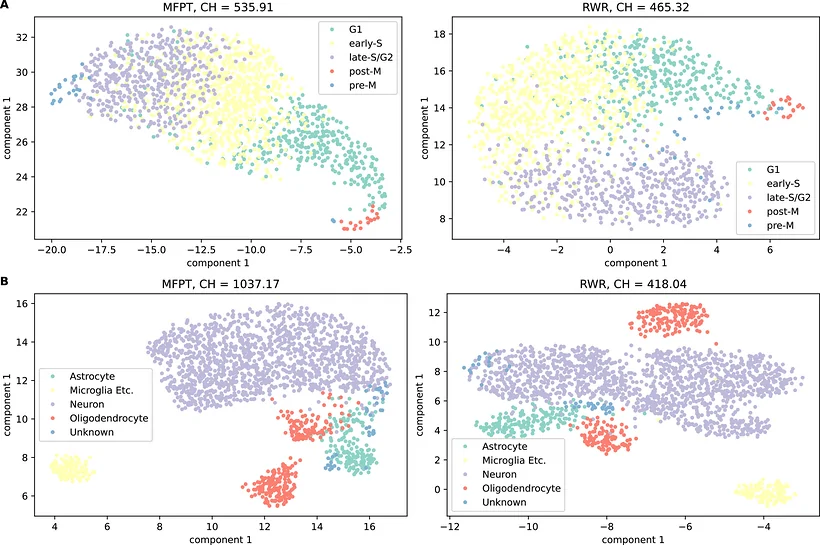
MFPT provides a biologically informative representation of sparse single‐cell Hi‐C data. (A) Low‐dimensional embeddings of the cell‐cycle dataset generated using the scHiCluster framework with MFPT transformation (left) or the original RWR‐based imputation (RWR; right). (B) Low‐dimensional embeddings of the mouse forebrain single‐cell Hi‐C dataset generated using the two representation strategies. The CH index of each embedding based on annotated cell type labels is indicated in the corresponding panel. In the mouse forebrain dataset, cells labeled as “Unknown” were excluded from CH index calculations.

Together, under the same downstream framework, MFPT achieved comparable clustering accuracy while producing embeddings that better preserved known biological relationships in both datasets. This controlled comparison directly demonstrates the advantage of the MFPT representation itself for sparse single‐cell Hi‐C analysis.

## 3 Discussion

In this study, we adapted the concept of mean first passage time (MFPT) from Markov chain theory to high‐throughput chromatin conformation capture data, thereby establishing a novel, graph‐distance‐based representation of contact matrices. This transformation provides a sequencing‐depth‐robust representation that preserves key topological features of chromatin organization. Building on these properties, we developed STRIDE, a library similarity metric that is robust to sequencing‐depth variation, technical noise, and experimental protocol differences, while preserving sensitivity to biologically meaningful differences in chromatin architecture. Furthermore, the MFPT representation can be integrated into sparse single‐cell Hi‐C analysis frameworks, providing a robust alternative to conventional imputation approaches.

Unlike conventional Hi‐C representations that rely directly on observed contact frequencies, the MFPT representation converts the contact matrix into a topological distance landscape derived from random‐walk dynamics on the interaction graph. By integrating information across multiple paths connecting genomic loci, MFPT captures the underlying connectivity structure rather than depending on individual observed contacts, allowing it to retain meaningful chromatin organization when direct contacts are sparsely sampled. This property distinguishes MFPT from existing smoothing or imputation approaches, which typically rely on predefined local neighborhoods [25] or limited random‐walk steps [23]. Moreover, because MFPT reflects effective topological distances rather than raw contact frequencies, its variation shows substantially weaker dependence on genomic distance, reducing the need for complex distance‐dependent normalization in downstream analyses [20]. Together, these properties suggest that MFPT provides a general representation framework for improving the robustness of sparse 3D genome analyses.

An intuitive analogy helps explain the robustness of MFPT to sequencing‐depth reduction. A Hi‐C contact matrix can be viewed as a weighted network, where genomic bins are connected by routes with different strengths determined by contact frequencies. During down‐sampling, weak connections corresponding to rare contacts are preferentially lost, whereas strong connections supported by frequent contacts are more likely to be preserved. Because MFPT measures effective distances by integrating random‐walk trajectories across the entire connectivity landscape, it is influenced primarily by the overall organization of high‐probability connections rather than by individual weak contacts. Therefore, the resulting distance relationships can remain stable under reduced coverage as long as the major connectivity patterns are retained.

MFPT is not without limitations. First, because MFPT integrates information across global connectivity patterns, it may introduce over‐smoothing at extremely high sequencing depths where raw contact maps already provide highly reliable local signals. This trade‐off reflects the fundamental difference between contact‐frequency‐based and topology‐based representations: MFPT prioritizes robustness to sparse sampling at the potential cost of reduced local contrast in deeply sequenced datasets.

Second, MFPT requires dense matrix operations and therefore does not preserve the sparse structure of the original contact matrix, which represents the major computational limitation of the approach. In this study, all analyses were performed on a workstation equipped with dual Intel Xeon Platinum 8380 processors (80 CPU cores) and 256 GB RAM. At whole‐genome 50‐kb resolution, STRIDE analysis required approximately 50 s of wall‐clock time (Table S9), with a peak memory usage of approximately 5.2 GB for human samples. Based on the observed scaling behavior, sequential chromosome processing at 5‐kb resolution is estimated to require a runtime on the order of 10 h and about 40 GB peak memory. These results indicate that MFPT/STRIDE remain computationally practical for bulk Hi‐C analyses at commonly used resolutions. In the single‐cell analyses presented in this study, MFPT transformation of approximately 2000 cells at 1‐Mb resolution required less than 5 min of wall‐clock time. However, scaling to millions of high‐resolution single‐cell Hi‐C libraries may require further optimization, including more efficient storage strategies and approximate computation schemes.

Third, as a graph‐based representation, MFPT relies on sufficient connectivity of the underlying contact network. In extremely sparse datasets where major connectivity patterns cannot be recovered, additional regularization or preprocessing may be required before MFPT calculation. Based on the datasets analyzed in this study, an empirical guideline is that approximately 20 million valid cis‐contact pairs are generally sufficient for human bulk Hi‐C data at resolutions of 10‐kb or coarser.

Finally, although extensive empirical evaluations in this study demonstrate that MFPT preserves biologically meaningful chromatin features and improves robustness to sequencing‐depth variation, a formal theoretical framework establishing the relationship between MFPT and the underlying spatial organization of chromatin remains to be developed. Future work integrating theoretical analysis with physical models of genome organization will help clarify the regimes in which MFPT most accurately reflects chromatin architecture. Moreover, MFPT should be interpreted as a graph‐based effective distance that measures the connectivity between bins through the chromatin interaction network. It cannot be directly interpreted or understood as a physical distance between bins.

Sequencing‐depth limitation is likely to remain a central challenge in 3D genomics for the foreseeable future, particularly as the field increasingly moves toward higher‐resolution, larger‐scale, and single‐cell measurements. Sparse sampling not only compromises the detection of local chromatin features such as TADs and loops [44, 45], but also complicates differential conformation analysis by increasing stochastic variation and false discoveries [46]. The robustness of MFPT representations across sequencing depths and technical perturbations suggests a potential new direction for addressing this long‐standing challenge. Although the downstream applications explored in this study represent initial demonstrations based on simplified analytical frameworks and are not yet fully optimized bioinformatics algorithms, MFPT‐based approaches consistently achieved performance comparable to, and in several cases exceeding, well‐established, optimized methods. These results support further development of MFPT‐centered computational strategies for a broader range of 3D genome analyses, including feature detection, differential analysis, and other tasks that remain robust to data sparsity.

The challenge of sparse measurements will become even more prominent with the continued expansion of single‐cell and spatial chromatin conformation technologies [16, 47]. Our results demonstrate that MFPT can provide an informative and depth‐robust representation of sparse single‐cell Hi‐C data, enabling biologically meaningful embeddings without relying on extensive task‐specific imputation procedures. Future work integrating MFPT representations with dedicated algorithms for single‐cell and spatial genomics may provide a general framework for extracting robust biological signals from increasingly sparse 3D genome datasets, including applications in developmental biology, disease‐associated heterogeneity, and cell‐state dynamics.

## 4 Conclusions

This study introduces a mean first passage time (MFPT)‐based representation for high‐throughput chromatin conformation capture data, transforming contact matrices into a topology‐aware distance representation. The MFPT representation shows empirically improved robustness to sequencing‐depth reduction while preserving biologically meaningful chromatin organization in the bulk Hi‐C, HiChIP, and single‐cell Hi‐C datasets analyzed. Building on this representation, we develop STRIDE, a library similarity metric that does not require dataset‐specific parameter tuning and enables robust comparison of chromatin conformation datasets across varying sequencing depths and experimental conditions.

Together, these results highlight the potential of MFPT‐based representations as a foundation for improving the analysis of sparse 3D genome data and motivate further development of MFPT‐based computational approaches for diverse chromatin conformation applications.

## 5 Methods

### 5.1 Calculation of MFPT Representation

The MFPT representation is derived from the mean first passage time (MFPT) theory of Markov chains. Given a symmetric, nonnegative intrachromosomal Hi‐C contact matrix *C*  =  {*c_ij_*}, the diagonal elements are first removed because self‐transitions do not represent contacts between distinct genomic loci and may dominate random‐walk dynamics. The resulting matrix is then normalized using the ICE style [48] matrix balancing procedure (bins with no contacts, together with the lowest 2% of bins ranked by coverage depth, are masked before balancing) to obtain a symmetric, balanced contact matrix *P*  = {*p_ij_*}_*n* × *n*_ ​, satisfying *p_ij_* ≥ 0,  ∀*i*,  *j* and $\sum_{j=1}^{n}p_{ij}=1,\;\forall i$, where *n* denotes the number of bins retained.

The matrix *P* can be interpreted as the adjacency matrix of an undirected graph taking the genomic bins as nodes. The random walk process on this graph can then be interpreted as a Markov chain taking *P* as the transition matrix. As long as the corresponding graph is connected, the random walk is irreducible and positive recurrent, thereby satisfying the theoretical requirements for MFPT computation. In practice, if this condition is not satisfied, we restrict the analysis to the largest connected component of the graph and recompute the balanced transition matrix on the corresponding contact submatrix. The resulting random walk process therefore captures the topological connectivity structure encoded by the Hi‐C contact map.

The first passage time, *M_ij_*, from bin *i* and *j* is defined as the number of steps required for a random walker starting from *i* to first reach *j*. The mean first passage time (MFPT) from *i* to *j* is then defined simply as

$$\;m_{ij}=E\left(M_{ij}\right)$$

By the properties of conditional expectation and the memoryless nature of the random walk process, the following results follows directly:

$$\;m_{ij}=\sum_{k\neq j}\left(1+E\left(M_{kj}\right)\right)p_{ik}+1\times \;p_{ij}=1+\sum_{k\neq j}p_{ik}m_{kj},\;\forall i,\;j$$

or expressed as the following matrix equation:

$$\left(I-P\right)M=E-P\times diag\left(M\right)$$

where *M*  = {*m_ij_*}_*n* × *n*_  and *E*  = {1}_*n* × *n*_.

The solution to this equation has been well‐studied [49], and one common method involves the introduction of the following auxiliary matrix

$$Z={\left(I-P+e\pi^{T}\right)}^{-1}\;$$

where *I* is the identity matrix, π represents the stationary distribution of the random walk process and *e*  = {1, 1, ···, 1}^*T*^  is a vector with unit elements. Then it has been shown that

$$M=\left(I-Z+E\times \mathrm{diag}\left(Z\right)\right)\;\times {\left(\mathrm{diag}\left(e\pi^{T}\right)\right)}^{-1}$$

Since *P* is a symmetric probability transition matrix, the vector *e* satisfies the equation for the stationary distribution, π × *P*  =  π. Therefore, it follows that π  =  *e*/*n*. Thus, the expression for *Z* can be simplified as:

$$Z={\left(I+\frac{1}{n}-P\right)}^{-1}\;$$

It is easy to prove that, for the symmetric transition matrix *P* defined above, *I* + 1/*n* − *P* is positive definite, ensuring that *Z* is well defined. The inversion of this symmetric matrix can be efficiently performed using Cholesky decomposition. Therefore, *M* can be calculated rapidly even at high resolutions.

The MFPT matrix obtained by this algorithm has two practical limitations. First, the diagonal elements are all equal to *n*, and most off‐diagonal elements are also close to *n*, resulting in a limited dynamic range. Second, the MFPT matrix is asymmetric because the expected time to reach bin *j* from bin *i* may differ from that in the reverse direction. However, Hi‐C contact frequencies describe reciprocal spatial proximity between genomic loci and are inherently symmetric. Therefore, the directional accessibility captured by MFPT needs to be converted into a symmetric representation.

For two genomic bins *i* and *j*, rapid transition from either direction indicates strong topological connectivity between these loci. We therefore used the smaller of the two directional MFPT values to represent their reciprocal connectivity:

$$MFPT_{\mathrm{ij}}=\frac{\min\;\left(m_{\mathrm{ij}},m_{\mathrm{ji}}\right)}{n}\;$$

This transformation produces a symmetric representation with strictly positive values and normalized diagonal elements equal to 1.

### 5.2 Determination of STRIDE Metric

For two Hi‐C libraries, we further restrict the calculation of MFPT to the largest set of bins for which all contact subgraphs involved in the comparison remain connected. Denoting their MFPT representations as *M*_1_ and *M*_2_, STRIDE quantifies their dissimilarity by comparing the relative differences between the log‐transformed MFPT matrices. The logarithmic transformation reduces the influence of the global dynamic range of MFPT values and emphasizes relative differences in chromatin organization. The STRIDE score is formally defined as:

$$\mathrm{STRIDE}\;=\frac{2∥\log\;\left(M_{1}\right)-\log\;\left(M_{2}\right)∥}{∥\log\;\left(M_{1}\right)+\log\;\left(M_{2}\right)∥}$$

where ∥ · ∥ denotes a matrix norm. The denominator normalizes the magnitude of the difference by the overall signal intensity of the two representations, allowing comparison between chromosomes with different numbers of bins and contact patterns. In this study, the Frobenius norm was used as the default norm for all bulk Hi‐C analyses. It should be noted that the choice of matrix norm, resolution, and normalization by the average signal magnitude only affects the scale of the STRIDE scores and has little impact on the inferred lineage relationships.

For bulk Hi‐C datasets, STRIDE was calculated chromosome‐wise using 50‐kb resolution matrices for human and mouse datasets and 10‐kb resolution matrices for the zebrafish dataset. These resolutions were selected to balance computational efficiency and the ability to capture biologically meaningful differences in chromatin organization.

Because library similarity scores can vary across datasets due to differences in chromosome size, genomic architecture, species, and matrix resolution, all benchmarking analyses were performed at the chromosome level rather than using averaged or trimmed summary scores. Furthermore, because the absolute STRIDE score depends on experimental conditions and analytical resolution, we do not define a universal threshold for distinguishing biological replicates from unrelated samples. Instead, STRIDE scores should be interpreted according to the experimental design and appropriate reference datasets.

### 5.3 Determination of TAD Boundaries Based on Insulation Score Profiles in Contact Maps

Insulation score (IS) profiles were calculated chromosome‐wise for both contact and MFPT maps following the configuration established in our previous study [30]. All analyses were performed at 10‐kb resolution using a 250‐kb neighborhood radius, corresponding to a local genomic scale of 50 bins. This window size was selected to capture domain‐scale insulation patterns while retaining sensitivity to local boundary variation [28].

For each chromosome and each representation, insulation scores were normalized by the chromosome‐wide average and log‐transformed to facilitate comparison across chromosomes and sequencing depths.

TAD boundary annotations were obtained exclusively from the complete‐depth contact maps, as described previously [30], and were used as fixed reference annotations throughout all down‐sampling analyses. Specifically, insulation score distributions within each chromosome were modeled using a three‐component Gaussian mixture model, representing strong boundaries, weak boundaries, and TAD interiors. Model parameters were estimated using the Expectation–Maximization (EM) algorithm, and each bin was assigned to the component with the highest posterior probability. Bins classified as boundaries (strong or weak) and corresponding to local insulation minima within a 21‐bin neighborhood were identified as candidate TAD boundaries. Adjacent candidate boundaries were merged into TADs when more than 50% of the intervening bins were classified as weak boundaries or TAD interiors.

### 5.4 Generation of Loop Position Annotations and Application of APA

Reference loops in GM12878 were defined using orthogonal chromatin interaction assays, including ChIA‐PET and HiChIP datasets. All published ChIA‐PET and HiChIP loop sets were downloaded from the corresponding original studies and lifted over to the hg19 genome assembly when necessary. The resulting loop sets were merged and converted to a common 10‐kb resolution. To obtain high‐confidence reference loops, loop strength was quantified using the fully sequenced GM12878 Hi‐C library, and loops with Z scores > 2 were retained (see the definition below). This procedure resulted in a final reference loop set containing 83,865 loops. Reference loop selection was performed independently of MFPT representations to enable unbiased comparison between contact maps and MFPT‐based representations. The corresponding negative interactions were defined as the genomic bin pairs whose two anchors were placed halfway between the corresponding anchors of two adjacent reference loops on the same chromosome with the same genomic span.

Loop strength Z scores and aggregate peak analysis (APA) were performed using identical procedures for contact maps and MFPT representations. Let *M* denote the map under evaluation. For each reference loop connecting bins *i* and *j* (*i* < *j*), local background statistics were estimated from two flanking regions:

$$M\left[\left(i-6\right):\left(i-3\right),\left(j-6\right):\left(j-3\right)\right]$$

and

$$M\left[\left(i+3\right):\left(i+6\right),\left(j+3\right):\left(j+6\right)\right]$$

The mean and standard deviation of signal values within these regions were denoted as *m* and *s*, respectively. The loop strength was then calculated as:

$$Z=\frac{M\left[i,j\right]-m}{s}\;$$

APA was performed by extracting normalized 13 × 13 local maps centered on all reference loops:

$$\frac{M\left[\left(i-6\right):\left(i+6\right),\left(j-6\right):\left(j+6\right)\right]-m}{s}$$

and averaging these normalized maps across all reference loops. The global APA score was calculated as the mean of *Z* scores at all the loop centers.

Because MFPT values represent transition times rather than contact frequencies, stronger chromatin connectivity corresponds to lower MFPT values. Therefore, Z scores calculated from MFPT representations were multiplied by − 1 before comparison with contact maps, ensuring that higher Z scores consistently represent stronger loop signals across both representations.

### 5.5 Pseudo‐Replication Generation and Down‐Sampling

Pseudo‐replicates of GM12878 libraries were generated by randomly partitioning the original Hi‐C contact reads into two equal‐sized sub‐libraries. Specifically, for a raw contact matrix *C*, the contact count of each bin pair was independently divided between the two pseudo‐replicates using binomial sampling:

$$c_{1ij}∼\mathrm{Binomial}\left(c_{\mathrm{ij}},0.5\right)$$

The second pseudo‐replicate was obtained as *c*_2*ij*_ = *c_ij_*  − *c*_1*ij*_, resulting in two independent contact matrices with approximately equal sequencing depths.

For down‐sampling analyses, contact reads were first grouped according to their genomic bin pairs. The grouped contacts were then merged into a single pool, from which a defined fraction of contacts was randomly sampled without replacement. The sampled contacts were subsequently used to reconstruct the down‐sampled contact matrices at the corresponding sequencing depths.

### 5.6 Application of Commonly Used Library Similarity Metrics

We benchmarked STRIDE against three widely used Hi‐C library similarity metrics: HiCRep [20], HiC‐spector [22], and genomeDISCO [23]. Although QuaSAR has also been evaluated frequently in previous studies [21], it was not included in our benchmark because its implementation is tightly coupled to the hifive package [50] and does not provide a standalone workflow for commonly used contact map formats such as plain text matrices or .hic files.

All similarity metrics were applied chromosome‐wise. Because similarity scores can vary substantially across chromosomes due to differences in chromosome size, contact density, and genomic architecture, we did not aggregate chromosome‐level scores into a single genome‐wide value. Instead, all evaluations were performed at the chromosome level. Unless otherwise specified, comparisons were conducted at 50‐kb resolution for human and mouse samples, and at 10‐kb resolution for zebrafish samples, which provide a balance between structural resolution and computational efficiency and have been widely used in Hi‐C benchmarking studies [23].

The HiCRep implementation was obtained from its GitHub repository (https://github.com/TaoYang‐dev/hicrep). The maximum genomic distance parameter was set to infinity to allow the full contact map to contribute to similarity estimation, ensuring consistency with other metrics. The smoothing parameter *h*, which controls the degree of matrix smoothing, was set to 5 at 50‐kb resolution and 20 at 10‐kb resolution for all datasets and chromosomes. These values were selected following the recommendation of the original authors for resolutions close to that used in this study, and their suitability was further confirmed by testing alternative values on representative chromosomes.

The HiC‐spector implementation was obtained from the GitHub repository associated with the original publication (https://github.com/gersteinlab/HiC‐spector) and applied using default settings. The number of eigenvectors was set to 20 according to the authors’ recommendation.

For genomeDISCO, the number of random‐walk transitions was set to 3 following the original publication, and the normalization method was set to “sqrtvc.”

Both HiCRep and genomeDISCO provide optional procedures for reducing sequencing‐depth differences through down‐sampling. These procedures were enabled consistently whenever applicable. HiC‐spector does not provide an equivalent depth‐normalization option; therefore, no additional normalization was introduced and the method was applied directly to the original contact matrices.

Because HiCRep, HiC‐spector, and genomeDISCO output similarity scores (where larger values indicate greater similarity), while STRIDE directly produces dissimilarity scores, all metrics were converted into comparable dissimilarity matrices before downstream analyses. For a similarity matrix *c*, the corresponding distance matrix was calculated as:

$$\;d_{ij}=\sqrt{2\left(1-c_{ij}\right)}\;$$

Hierarchical clustering and Calinski–Harabasz (CH) index calculations were performed using the resulting dissimilarity matrices rather than the original similarity scores.

### 5.7 Differential Chromatin Contact (DCC) Analysis Based on the MFPT Representation

DCC analysis is performed based on loop Z scores calculated from MFPT representations at 10‐kb resolution. For two conditions under comparison, *M*_1_ and *M*_2_ denote the log‐transformed MFPT representations, and *Z*_1_ and *Z*_2_ denote the corresponding MFPT‐derived loop Z‐score matrices, respectively, following the definition described above. The corresponding balanced contact frequency matrices are denoted as *P*_1_ and *P*_2_. Given four significance parameters *p*_1_, *p*_2_, *p*_3_, and *p*_4_, a bin pair (*i*, *j*) is identified as a condition 1‐induced DCC if all of the following criteria are satisfied:

1) The interaction shows significant enrichment in condition 1 according to the MFPT representation:

$$Z_{1}\left[i,j\right]>\phi_{\sigma =1}\left(1-p_{1}\right)$$

2) The MFPT‐derived interaction strength differs significantly between the two conditions:

$$Z_{1}\left[i,j\right]-Z_{2}\left[i,j\right]>\phi_{\sigma =\sqrt{2}\;}\left(1-p_{2}\right)$$

3) In the original contact frequency representation, the central region of the candidate interaction is significantly enriched in condition 1. Specifically, the Mann–Whitney U test comparing:

$$\left\{P_{2}\left[i+k,j+l\right]|k,l\in \left[-1,1\right]\times \left[-1,1\right]\right\}$$

with:

$$\left\{P_{2}\left[i+k,j+l\right]|k,l\in \left[-6,-3\right]\times \left[-6,-3\right]\cup \left[3,6\right]\times \left[3,6\right]\right\}$$

returns a *p*‐value smaller than *p*_3_.

4) The corresponding interaction is not enriched in condition 2. The Mann–Whitney U test comparing the same central and background regions in *P*_2_ returns a *p*‐value greater than 1 − *p*_4_.

5) The candidate interaction represents the strongest MFPT signal within its local neighborhood:

$$M_{1}\;\left[i,j\right]=\min_{k,l\in \left[-6,+6\right]}M_{1}\left[i+k,j+l\right]\;$$

where $\phi_{\sigma }\left(*\right)$ denotes the quantile function of a normal distribution with mean 0 and standard deviation σ. Condition 2‐induced DCCs are identified using the same procedure with the two conditions reversed.

These criteria ensure that a condition 1‐induced DCC satisfies three complementary requirements: (i) significant interaction enrichment in the MFPT representation under condition 1; (ii) significant interaction strength differences between the two conditions; and (iii) consistent enrichment patterns in the original contact frequency representation, where local contact enrichment is present in condition 1 but absent in condition 2. The local minimum criterion in the MFPT representation ensures that only the most prominent differential contact loci within each neighborhood are reported, avoiding redundant reporting of extended differential regions.

The following thresholds are used throughout this study: *p*_1_ =  0.01,   *p*_2_ =  0.01,   *p*_3_ =  0.001,   *p*_4_ =  0.1. Following previous studies, only DCCs with genomic distances between 70 kb and 5 Mb are reported.

HiCcompare is performed following the workflow recommended in the original publication [36]. For both DCC analyses, the significance threshold applied to HiCcompare results is adjusted to obtain a number of reported DCCs comparable to that obtained using the MFPT‐based method.

### 5.8 MFPT‐Based Single‐Cell Hi‐C Data Analysis

All MFPT‐based single‐cell Hi‐C analyses in this study are performed at 1‐Mb resolution. The analytical framework follows the overall architecture of scHiCluster [25], with modifications introduced only in the representation and imputation steps. Therefore, only the differences from the original scHiCluster workflow are described below.

During the Gaussian smoothing step, the kernel size is expanded from the default 3 × 3 to 7 × 7. This modification is introduced to ensure the connectivity of the contact graph required for MFPT calculation across all single cells. The filtering of bins according to coverage depth during matrix balancing is skipped and all bins participate in the calculation of MFPT. The expanded smoothing window has minimal impact on the performance of the original scHiCluster framework.

In the imputation step, the original random‑walk‑with‑restart (RWR)‑based imputation is replaced by the MFPT transformation, followed by a logarithmic transformation. The resulting matrix is then further normalized by its Frobenius norm. Furthermore, the binarization step used in the original scHiCluster workflow is removed. Instead, the upper triangular elements of the log‑transformed MFPT representation of each cell are directly flattened and used as input features for singular value decomposition (SVD), without additional preprocessing, feature selection, or filtering.

## Author Contributions

B.X.X., Z.H.Z., and F.F.L. conceived this project. B.X.X. and F.F.L. analyzed data. B.X.X., F.F.L., W.C.T., and Z.H.Z. prepared the manuscript. X.M.G. prepared the figures and tables.

## Funding

This work was supported by the National Natural Science Foundation of China (32200515 to BXX, 32470672 to FFL and 32341011, 32550326 to ZHZ), the Hebei Natural Science Foundation (C2026202008 to BXX), the Strategic Priority Research Program of CAS (XDA0460300, XDC0200000 to ZHZ), the Biological Breeding‐National Science and Technology Major Project (2022ZD04017 to ZHZ), the Natural Science Foundation of Guangdong Province (2025A1515012339 to FFL) and Guangdong Provincial Medical Research Fund (A2026300 to FFL).

## Conflicts of Interest

The authors declare no conflicts of interest.

The code in this study is hosted on Gitee and freely accessible at the URL https://gitee.com/matrix_evolution/STRIDE. The STRIDE package is freely available at PyPI following the URL https://pypi.org/project/hicstride.

## Supporting information




**Supporting File 1**: advs78529‐sup‐0001‐SuppMat.docx.

**Supporting File 2**: advs78529‐sup‐0002‐SuppData.zip.

## Data Availability

The datasets analyzed during the current study are available in the gene expression omnibus (GEO) repository, the ENCODE project [51] and the genome sequence archive (GSA) in the National Genomics Data Center [52]. The detailed information together with the sequencing depths and sparsity indices of the libraries involved are listed in Table S1.
