# Supplementary material for: STRIDE: A Sequencing Depth Robust Metric for Sparse Chromosome Conformation Capture Data Comparison
Source: Adv Sci (Weinh). 2026 Oct 28:e78529. Online ahead of print. doi: 10.1002/advs.78529 (PMC13715917; doi:10.1002/advs.78529)
Supplement: Supplementary file 1 [file ADVS-9999-e78529-s002.docx]

**STRIDE: A Sequencing Depth Robust Metric for Sparse Chromosome Conformation Capture Data Comparison**

Bingxiang Xu ^1,2,^*, Xiaomeng Gao ^3^, Weichu Tao ^2^, Zhihua Zhang ^4,5,6,^*, Feifei Li ^7,^*

1, Key Laboratory of Hebei Province for Molecular Biophysics, Institute of Biophysics, School of Health Science & Biomedical Engineering, Hebei University of Technology, Tianjin, 300130, China

2, School of Exercise and Health, Shanghai University of Sport, Shanghai, 200438, China.

3, Department of Cellular and Molecular Medicine, University of California, San

Diego School of Medicine, La Jolla, CA, USA

4, China National Center for Bioinformation, Beijing, China

5, Beijing Institute of Genomics, Chinese Academy of Sciences, Beijing, China

6, School of Life Science, University of Chinese Academy of Sciences, Beijing, China.

7, Division of Cell, Developmental and Integrative Biology, School of Medicine, South China University of Technology, Guangzhou 510006, China.

*: These authors jointly supervised this work, xubingxiang@sus.edu.cn (XBX); zhangzhihua@big.ac.cn (ZZH) and liff@scut.edu.cn (LFF).

# Public data analysis.

All public Hi-C datasets employed in this study are enumerated in (Table S1). The quality of the downloaded sequencing reads was assessed using FastQC (accessible at https://github.com/s-andrews/FastQC). Reads exhibiting substandard sequencing quality (quality score $\leq10$) and those containing adaptor sequences were meticulously purged utilizing the fastp tool. Subsequently, the refined reads were channeled through the HiC-Pro pipeline, adopting its default parameters for processing. To maintain consistency with their source publications, the hg19 human reference genome was used for the GM12878, K562, hESC, and IMR90 datasets.

For matrix equilibration, the Kilian-Rajagopalan (KR) algorithm was employed. Initially, all diagonal entries within the contact matrix were set to zeros. This step preceded the exclusion of bins with extremely low sequencing coverage, specifically targeting those whose row sums were below the second percentile of row sums from bins with positive row sums. Upon reaching convergence, the matrix was further normalized so that both row and column summations were rigorously normalized to a unit value. Let $s_{i}$ represent the row sum of bin $i$ in the balanced matrix $\{w_{ij}\}$, and let $m$ denote the maximum among all $s_{i}$ values. The elements $w_{ij}$ were then adjusted according to the formula:

$$w_{ij}=\left\{ \begin{aligned} w_{ij}/m, &i\neq j \\ 1-s_{i}/m, &i=j \end{aligned} \right..$$

# Calculating the signal to noise of contact frequencies or MFPT maps.

The signal-to-noise ratio (SNR) was computed based on either KR-normalized contact frequencies or mean first passage time (MFPT) values. In this context, data from fully sequenced libraries were considered as the 'clean' signals, while those from down-sampled libraries represented signals contaminated with noise. The analysis was conducted on maps with a resolution of 10 kilobases (Kb). Only genomic regions with distances not exceeding 2 megabases (Mb) were included in the analysis. For contact frequency calculations, regions exhibiting zero values in either the fully sequenced or down-sampled libraries were preemptively excluded. Subsequently, the values were subjected to a log2 transformation. A linear regression analysis was then performed, wherein the transformed values from the down-sampled libraries were regressed against those from the fully sequenced libraries. The SNR was subsequently defined as the ratio of the regression sum of squares to the residual sum of squares derived from the regression model. This computation was executed separately for each chromosome.

# Calculating the genome distance for the variability of contact frequencies (MFPTs) to stabilize.

For a (chromosome-wise) contact map or MFPT map at 10Kb resolution $\left\{ c_{ij} \right\}_{n\times n}$, the coefficient of variation (CV) of the $\log_{2}$ transformed contact frequencies or MFPT values along the same genome distances were determined as

$$cv_{d}=\frac{std(\{c_{ij}|j-i=d\})}{mean(\{c_{ij}|j-i=d\})},d=1,2,\cdots300.$$

Then, the genomic distance at which the CV values reached their stability was determined as the abscissa of the point where the curve $cv(d)$ tangent to the line with slope $k=\frac{cv\left( 300 \right)-cv(1)}{299}$ among all lines with that slope.

# Calculation of the intra- and inter- TAD average contact frequencies or MFPTs around TAD boundaries.

For a contact frequency or MFPT map at 10Kb resolution $\left\{ c_{ij} \right\}_{n\times n}$ and a TAD boundary $i$, the intra- and inter-TAD average contact frequency or MFPT was calculated as follows:

$$\left\{ \begin{aligned} \mathrm{intra}=\frac{1}{1640}(\sum_{k=i-50}^{i-5} \sum_{l=k+5}^{i-5} c_{kl}+\sum_{k=i+5}^{i+45} \sum_{l=k+5}^{i+50} c_{kl}) \\ \mathrm{inter}=\frac{1}{3016}(\sum_{k=i-50}^{i+45} \sum_{l=k+5}^{i+50} c_{kl}-\mathrm{intra}) \end{aligned} \right..$$

# De novo TAD detection from the MFPT representation.

The de novo TAD-detection procedure was adapted and simplified from our previously described framework. Briefly, candidate TAD boundaries were first identified from the insulation-score profile, and the genomic intervals between adjacent candidate boundaries were subsequently evaluated according to the shape and strength of their insulation profiles.

For a given chromosome, let

$$S=\left\{ s_{i} \right\}_{i=1}^{N}$$

denote the negative insulation-score profile derived from the MFPT representation, where $N$ is the number of genomic bins. A bin $i$ was defined as a candidate TAD boundary if $s_{i}$ was a strict local minimum within a $\pm10$-bin window, i.e., within the interval$[i-10,i+10]$. The genomic interval between two adjacent candidate boundaries was then defined as a candidate TAD.

For each candidate TAD $\left[ a , b \right]$, the relationship between $S$ and genomic position within the interval was independently fitted using a linear model and a quadratic model. An *F*-test was used to determine whether the quadratic model provided a significantly better fit than the linear model, and the resulting $P$-value was denoted as $p_{1}$. If the maximum of the fitted quadratic curve occurred at genomic bin $m$, its relative position within the candidate TAD was defined as

$$r=\frac{m-a}{b-a}.$$

This criterion was used to ensure that the characteristic peak of the insulation profile was located within the central region of the candidate TAD rather than near either boundary.

We further quantified the prominence of the insulation signal within each candidate TAD as

$$v_{\left[ a , b \right]}=\max_{a<i<b}s_{i}-\max\left( s_{a} , s_{b} \right).$$

To estimate the significance of $v_{\left[ a , b \right]}$, a normal distribution was fitted by least squares to the $1/30, 2/30,\ldots,10/30$ quantiles of the genome-wide distribution of $v_{\left[ a , b \right]}$. Let $\Phi$ denote the cumulative distribution function of the fitted normal distribution. For each candidate TAD, a one-sided empirical significance measure was calculated as

$$p_{2}=1-\Phi\text{ }\left( v_{\left[ a , b \right]} \right).$$

A candidate interval $\left[ a , b \right]$ was retained as a high-confidence TAD only when all of the following criteria were satisfied: $p_{1}<0.01,$ $\mid r-0.5\mid\leq0.3,$ and $p_{2}<0.05.$ The two endpoints, $a$ and $b$, of each retained interval were reported as TAD boundaries.

# De novo loop detection from the MFPT representation.

Following the notation used in the main text, for a given chromosome at 10-kb resolution, let $C$ denote the contact-count matrix, $M$ the corresponding MFPT representation, and $Z$ the loop Z-score matrix derived from $M$. A bin pair $\left( i , j \right)$, with $i<j$ and $6<j-i<200$, was initially defined as a candidate loop if it satisfied both $Z_{ij}<\phi_{0.01}$ and

$$M_{ij}=\min_{\begin{matrix} k\in\left[ i , -6i+6 \right] \\ l\in\left[ j , -6j+6 \right] \end{matrix}}M_{kl},$$

where $\phi_{0.01}$ was the $0.01$ quantile of standard normal distribution. Thus, candidate loops correspond to locally strong interaction signals in the MFPT representation within genomic distances of approximately 70 kb to 2 Mb.

Because the smoothing effect of MFPT may slightly shift the apparent position of a loop, candidate loop coordinates were further refined using the corresponding contact-frequency matrix. Let $O$ denote the observed/expected contact matrix obtained by balancing $C$ and subsequently normalizing each diagonal by its mean contact frequency. For each candidate loop $\left( i , j \right)$, the position of the maximum value of $O$ within $\left[ i-6,i+6 \right]\times[j-6,j+6]$ was identified. The candidate was retained only when this local maximum fell within $\left[ i-2,i+2 \right]\times[j-2,j+2]$*,* indicating that the interaction peak in the contact map was spatially consistent with the MFPT-derived candidate. The loop coordinate was then reassigned to this local maximum, and the refined coordinate was again denoted by $\left( i , j \right)$.

The statistical significance of local contact enrichment was then evaluated using the original contact-count matrix $C$. For each refined candidate loop, we defined

$$m=\sum_{\left( k , l \right)\in\left[ i-1,i+1 \right]\times[j-1,j+1]} C_{kl},$$

corresponding to the total contact count in the central $3\times3$ window. We further defined

$$m_{0}=\sum_{\left( k , l \right)\in R} C_{kl},$$

where $R=\left[ i-6,i-4 \right]\times\left[ j-6,j-4 \right]\bigcup\left[ i-1,i+1 \right]\times\left[ j-1,j+1 \right]\bigcup\left[ i+4,i+6 \right]\times\left[ j+4,j+6 \right]$. These three equally sized $3\times3$ regions represent the central loop signal together with flanking background regions. Under the null hypothesis that contacts are distributed equally in the central and flanking regions, the expected probability of a contact falling within the central region is $1/3$. The enrichment significance of the candidate loop was therefore calculated using a binomial model:

$$p=1-F_{\mathrm{Binom}\left( m_{0} , \text{ }1/3 \right)}\left( m \right),$$

where $F_{\mathrm{Binom}\left( m_{0} , \text{ }1/3 \right)}$denotes the cumulative distribution function of a binomial distribution with $m_{0}$ trials and success probability $1/3$. To avoid unstable or extreme significance estimates in very low-contact regions, only candidate loops with $m_{0}>20$ were subjected to statistical testing.

Finally, the resulting $P$-values were adjusted for multiple testing using the Benjamini–Hochberg procedure. Candidate loops with $\mathrm{FDR}<0.05$were reported as significant loops, and loops were ranked according to their nominal $P$-values.

# Calculation of sequencing-depth sensitivity and discrimination indices for library-consistency metrics.

For a series of down-sampling fractions, suppose that the function of consistency scores against the $\log_{2}$ transformed sampling rate for PR, BR and NR were denoted as $p\left( d \right), b\left( d \right)$ and $n\left( d \right)$ where $d=0,1,2,\cdots,5$. Scores were firstly transformed into the $\left[ 0,1 \right]\times\left[ 0,1 \right]$ square by taking the following transform

$$f^{'}\left( d^{'} \right)=f(5\times d)/max(n\left( 5 \right),$$

where $f$ represented one of $p$, $b$ or $n$, $d^{'}$=$\frac{d}{5}$. Then, each of these functions was fitted with a 5th-order polynomial where all coefficients were non-negative. The results of the fitting were still denoted as $p^{'}$, $b^{'}$ or $n^{'}$. The sequencing depth sensitivity index was then calculated as

$$\int_{0}^{1} f^{'}\left( t \right)dt-f\left( 0 \right),$$

where $f=p, b \mathrm{or} n$.

The discrimination indices between PR and BR, as well as between BR and NR, were calculated as the roots of the equations $p^{'}\left( t \right)=b\left( 0 \right)$ and $b^{'}\left( t \right)=n\left( 0 \right)$, respectively.

# Extended Data Figure

**Fig. S1 Advantages of the MFPT representation over raw contact maps.** **(A)** Toy example illustrating how MFPT recovers structural information lost due to low sequencing depth. Ten equally spaced 10-kb bins from chr6: 90.00–100.00 Mb in the GM12878 dataset were used. Contact maps are shown as weighted transition graphs. At low depth, weak or rare contacts drop to zero and edges disappear (middle). MFPT restores effective distances between disconnected bins by averaging the lengths of indirect paths through intermediate nodes (right), thereby mitigating the effects of down-sampling. **(B)** Relationship between coefficient of variation (CV) of contact frequencies (left) or MFPT values (right) and genomic distance for chromosomes other than chromosome 6 (shown in Fig. 1E, F). For contact frequencies, zero-count bin pairs were excluded from CV calculation. Markers and color coding are identical to Fig. 1E, F.

**Fig. S2 MFPT representation preserves TAD and loop features more robustly than conventional contact maps in low-coverage datasets. (A, B)** Average contact-frequency profiles centered on annotated TAD boundaries across all down-sampling levels derived from conventional contact maps (A) and Raichu-normalized maps (B). **(C)** Consistency of de novo TADs identified from MFPT representations and conventional contact maps across progressively reduced sequencing depths. **(D)** Percentage of TAD boundaries overlapping ChIP-seq peaks of boundary-associated factors, including CTCF, RAD21, and SMC3, at each sequencing depth. Two TADs are considered consistent only when their overlapping region accounts for >80% of the length of both TADs. **(E, F)** Aggregate peak analysis (APA) of conventional contact maps (E) and Raichu-normalized maps (F) across all down-sampling levels. Only reference loops spanning >150 kb were included. Numbers indicate the average loop Z-score across all analyzed loops. **(G)** ROC curves and corresponding AUC values for distinguishing validated loops from matched negative interactions using loop Z-scores derived from the MFPT representation, conventional contact maps, and Raichu-normalized maps. **(H, I)** Contact profiles centered on a validated contact between the promoters of CCDC58 and FAM162A (supported by H3K27ac HiChIP) derived from conventional contact maps (H) and the MFPT representation (I) across all down-sampling levels. Corresponding loop Z-scores are indicated. **(J)** Ablation curves showing that MFPT supports robust de novo chromatin-loop identification across sequencing depths. Loops are independently identified from MFPT representations using the simplified MFPT-based caller, or from conventional balanced contact maps and Raichu-normalized maps using mustache. For all three approaches, loops are filtered at an FDR threshold of 0.05 and ranked from the most to the least significant. At each sequencing depth, the cumulative number of loops supported by the independent ChIA-PET and HiChIP reference set is plotted against the number of top-ranked loops considered. A predicted loop is considered matched to a reference loop when the distance between their loop centers is ≤20 kb, following the criterion used in the mustache study.

**Fig. S3 Dissimilarity scores for pseudo-replicates (PR), biological replicates (BR), and non-replicates (NR) computed by STRIDE, HiCRep, HiC-spector, and genomeDISCO across all down-sampling fractions in chromosomes 2–22 and X.** (Chromosome 1 is shown in Fig. 3A; shaded areas indicate 95% confidence intervals derived from 10 independent down-sampling trials.

**Fig. S4 STRIDE provides consistent and robust library similarity measurements across sequencing depths. (A)** Sequencing-depth sensitivity score, using HiCRep dissimilarity values for chromosome 1 as an example. The score quantifies the rate at which dissimilarity increases with progressive down-sampling (see Supplementary Text). **(B)** Discrimination index among pseudo-replicates (PR), biological replicates (BR), and non-replicates (NR), using HiCRep results for chromosome 16 as an example. The index measures the amount of down-sampling noise required to collapse meaningful biological distinctions (see Supplementary Text).

**Fig. S5 Reference biological relationships and the visualization of the library (dis)similarity matrices (by MDS projection into two-dimension spaces) of the bulk Hi-C and HiChIP datasets analyzed in this study. (A)**, **(F)**, and **(H)** show the reference relationships used for the hESC/IMR90, zebrafish HSPC developmental, and early porcine embryonic developmental datasets, respectively. (B), (C), (D), (E) and (G) show the visualization of the library (dis)similarity matrices hESC/IMR90 dataset, independently generated GM12878 and K562 cell dataset (chr1), HiChIP datasets of H3K27ac in K562 against GM12878 cells (chr21), the HiChIP datasets of cohesin and H3K27ac in mESC cells (chr9), and the zebrafish HSPC developmental dataset (chr6). The coordinates for each metric were scaled to the same plotting range to facilitate visual comparison. The lines indicate the LDA-derived decision boundaries taking the cell type or library type as the class labels.

# Extended Data Table Legends

**Table S1.** Summary of the datasets used in this study. The table includes effective sequencing depths (defined as the number of valid cis contacts), sparsity, and the number of retained bins after matrix balancing for all bulk Hi-C libraries used in the STRIDE benchmark at the resolutions used for each analysis.

**Table S2.** Raw similarity scores of STRIDE, HiCRep, genomeDISCO, and HiC-spector for PR, BR, and NR comparisons across progressive down-sampling levels in the GM12878 and K562 datasets. Columns 0–5 correspond to the full sequencing depth and down-sampling rates of 1/2, 1/4, 1/8, 1/16, and 1/32, respectively.

**Table S3.** The chromosome wise scores of the metrices compared with STRIDE in comparing the hESC and IMR90 cells. The results of the hierarchical clustering are also attached in the sheet named as “clustering”. The visualization of all library (dis)similarity matrices by MDS projection to two-dimensional spaces are attached in the sheet named as “MDS”.

**Table S4.** The chromosome wise scores of the metrices compared with STRIDE in the independently generated GM12878 and K562 dataset. The results of the hierarchical clustering are also attached in the sheet named as “clustering”. The visualization of all library (dis)similarity matrices by MDS projection to two-dimensional spaces are attached in the sheet named as “MDS”.

**Table S5.** The chromosome wise scores of the metrices compared with STRIDE in comparing the HiChIP datasets of H3K27ac in K562 against GM12878 cells. The results of the hierarchical clustering are also attached in the sheet named as “clustering”. The visualization of all library (dis)similarity matrices by MDS projection to two-dimensional spaces are attached in the sheet named as “MDS”.

**Table S6.** The chromosome wise scores of the metrices compared with STRIDE in comparing the HiChIP datasets of cohesin and H3K27ac in mESC cells. The results of the hierarchical clustering are also attached in the sheet named as “clustering”.

**Table S7.** The chromosome wise scores of the metrices compared with STRIDE in the zebrafish HSPC developmental dataset. The results of the hierarchical clustering are also attached in the sheet named as “clustering”. The visualization of all library (dis)similarity matrices by MDS projection to two-dimensional spaces are attached in the sheet named as “MDS”.

**Table S8.** The chromosome wise scores of the metrices compared with STRIDE in the early porcine embryonic development dataset. The results of the hierarchical clustering are also attached in the sheet named as “clustering”.

**Table S9.** Running time comparison of STRIDE, HiCRep, genomeDISCO, and HiC-Spector across all real Hi-C datasets analyzed in this study. For each method, the computation time required to calculate the similarity / dissimilarity score for every pair of cell types / stage is reported, along with the average time per pair. All evaluations were performed with sufficient CPU cores and memory allocated to enable parallel processing of all chromosomes simultaneously. Contact maps were pre-loaded into memory prior to scoring, thereby excluding I/O time from the measurements.
